# Influence of halloysite nanotubes on the efficiency of Asparaginase against mice Ehrlich solid carcinoma

**DOI:** 10.1016/j.sjbs.2022.02.058

**Published:** 2022-03-04

**Authors:** B.M.M. Baharoon, A.M. Shaik, Salim M. El-Hamidy, Rady Eid El-Araby, Ashwaq H. Batawi, Mohamed Abdel Salam

**Affiliations:** a Department of Biological Sciences, Faculty of Sciences, King Abdulaziz University, Jeddah, Saudi Arabia; b Princess Dr. Najla Bint Saud Al Saud Center for Excellence Research in Biotechnology, King Abdulaziz University, Jeddah, Saudi Arabia; c Central Lab, Theodor Bilharz Research Institute (TBRI) Ministry of Scientific Research, Egypt; d Department of Chemistry, Faculty of Sciences, King Abdulaziz University, Jeddah, Saudi Arabia

**Keywords:** Halloysite nanotubes, Cancer, Asparaginase, Liver, Histopathology, Liver functions, IP, Intraperitoneal, ALB, Albumin, ALP, Alkaline Phosphatase, ALT, Aniline Aminotransferase, ASNase, Asparaginase, AST, Aspartate Aminotransferase, BCP, bromocresol purple, BD, Bile Duct, CV, Central Vein, DDS, Drug Delivery Systems, EAC, Ehrlich ascites carcinoma, ESC, Ehrlich Solid Carcenoma, HNTs, Halloysite Nanotubes, IFCC, international federation of clinical chemistry, IM, Intramuscularly, IT, Intratumorally, KAU, King Abdulaziz University, KFMRC, King Fahd Medical Research Center, PV, Portal Vein, TBIL, Total Bilirubin, TEM, Transmission Electron Microscope, TP, Total protein, XRD, X-Ray Diffraction

## Abstract

Herein, the impact of the halloysite nanotubes to suppress the side effects of Asparaginase (ANase) cellular proliferation was investigated. Methods: A total of 100 adult male mice was employed. These mice were divided into four equal groups; Group 1 (control), Group 2 (ESC group) of a single dose of 0.15 ml Ehrlich cells (2 × 10^6^) intraperitoneal infusion(IP), Group 3 (ESC + ANase group) received six doses equal treatments of Intratumoral (IT) 0.07 ml Aspragnase (7 mg/kg) over two weeks. For two weeks, Group 4 (ESC + ASNase + HNTs) received an IT administration of 0.07 ml Asparaginase stocked on Halloysite nanotubes (HNTs) (30 mg/kg) three times per week. A blood specimen was collected, and the liver was removed to be investigated histologically. Results: TEM measurements for the Halloysite nanoclay showed their tubular cylindrical shape with a mean diameter of 50 nm and an average length of 1 μm, whereas The X-ray diffraction pattern of the Halloysite nanoclay showed their characteristic peaks. ESC increases the serum levels of aspartate aminotransferase, alanine aminotransferase, alkaline phosphatase, and bilirubin than control and other groups, even as albumin and total protein were decreasing. After using Halloysite Nanotube, the rates of these variables were enhanced up to 75%. The hepatocytes histological studies showed protection against Ehrlich Solid carcinoma-induced degenerative, necrotic, and inflammatory changes up to 70%. In conclusion, halloysite nanotubes have demonstrated effective removal of Ehrlich solid carcinoma in mice using an ASNase delivery system. It promoted the ASNase to inhibit the adverse effect of ANase's on the liver and remove the tumour cells.

## 1 Introduction

Nanotechnology has shown great attention as an advanced and promising technology in nanomedicine, such as cancer treatment, drug delivery/ release, and bio imaging. The main advantage of nanomaterials is that their small size enables them to penetrate the cell membrane and consequently can be used as efficient nanocarriers for drugs (Kopeckova et al., 2019). The bioavailability (Zhen et al., 2014) and therapeutic superiority (Mo et al., 2014) are essential factors to improve the pervasion and retention effects (Fang et al., 2016, Misra et al., 2010, Aslan et al., 2013). However, nanotubes for tumour treatment are more beneficial than traditional techniques due to their ability to treat the infected part of the body without affecting the healthy parts and avoid the conventional side effects (Parveen and Sahoo, 2008, Yang et al., 2015, Wright, 2014).

Nanotherapeutics and nanopharmaceuticals have the potential to enable and promote previous and more concise personal diagnoses, help increase treatment strategies, minimize adverse effects, and improve treatment tracking. These benefits will enhance the quality of life for people, promote better health and a much more self-reliant elderly population, and help medical care become more price (Wu et al., 2020).

Hepatocytes are considered a major detoxifying center for damaging toxic substances and medications, shielding them from adverse effects. The liver plays a crucial role in the detoxification method (Mescher, 2013). According to one of the results; the compounds of these toxins frequently caused cell injury in the liver. The most prevalent biochemical parameters of liver injury are AST and ALT (Arakawa et al., 2016, Shin et al., 2016). The tumour is the main reason for death in the twenty-first century all around the globe (Hashem et al., 2020). In 2015; 16; 210 cases of humans infected with cancer were recorded by the Saudi Cancer Registry (Alrawaji et al., 2015).

Ehrlich ascites carcinoma (EAC) is a category of amorphous melanoma marked by rapid proliferation, elevated transplant ability, and a short survival time (Ozaslan et al., 2011). Because EAC resembles human cancer cells, the solid and ascetic patterns of melanoma are commonly utilized to determine the anticancer effects of various metals (Da Silva et al., 2009, Kabel et al., 2013). Cancerous cells have a much higher metabolic rate than healthy cells, have a more excellent prevalence ratio, and withstand cell death paths such as apoptosis (Abbaszadeh et al., 2020). Indistinct solid tumour, such as solid Ehrlich carcinomas, are commonly used for cancer investigation. It was used in cancer model development and chemotherapy research (Silva et al., 2006). Chemotherapy agents, like conventional medicine, have unfavorable impacts on the body's standard organ systems (Nurgali et al., 2018). Liver cell injury is an example of adverse effects that can be disastrous and crucial to human health (Renu and Arunachalam, 2018).

Asparaginase is an enzyme being used in food production and medication. Asparaginase works by breaking asparagine, an amino acid that cancer cells can't make protein without (Fatima et al., 2019, El-Gendy et al., 2021).

Drug delivery systems (DDS), those with tracking assets, could be a viable solution to specific issues, as DDS can get extra security, good stability, and therapeutic efficacy of payloads (Lin et al., 2013). Even though DDS is not commonly used throughout medical therapy, various investigations are currently being utilized to evaluate the potential advantages of DDS for Provides regulated (Oluwadamilola et al., 2017). Most of these DDS have demonstrated a desirable, beneficial influence in vivo and in vitro, indicating that they have good medical potential (Yu et al., 2020). The composition of a DDS is influenced by both the method of preparation and the conformer used. Because of their notable accomplishments with medication atoms, reliability, cramping limit, wealth, and common harmful effects, mud metals have been suggested as functional MDDS additives (Carazo et al., 2018, Fakhrullina et al., 2015, Jafarbeglou et al., 2016, Sandri et al., 2016).

Because they target cancer cells while causing actual damage to healthy tissues, nanoparticles as therapeutic agents are an appealing appropriate treatment modality (Vasir et al., 2005).

Halloysite nanotubes (HNTs) are naturally occurring tubular clay used to implement medicines and drug delivery (Marinelli et al., 2021). In contrast to polymers, HNTs have a lot of promise, and research into halloysite nanotubes-drug interactions could lead to the development of clay-based drug delivery applications (Sandri et al., 2020). HNTs is a potential platform for drug transfer, polymer pitch, and adsorbents for environmental and biotechnological fields, according to researchers' successes over the last ten years. To increase the bioactivity and hydrophilicity of HNTs, it is necessary to change their surface (Danyliuka et al., 2020). HNTs are unique to nature's maximum fascinating and accessible nanomaterials. They have the same chemical composition as kaolin clay. HNTs' entire empty tube structure has a unique combination of remarkable features, including excellent mechanical, thermal consistency, high density as a method of usability, biocompatibility, and low cost (Emad et al., 2019). HNTs are great as filler materials for polymers and as containers for bioactive constituents (Shchukina and Shchukin, 2019, Cavallaro et al., 2018), leading to their practical applications in medicine, tumour cell separation, and tissue repair (Liu et al., 2019, Li et al., 2019,; Kryuchkova et al., 2020).

Carbon and clay nanotubes are actively competitive nanoparticles (Rozhina et al., 2021, 106041https://doi.org/10.1016/j.clay.2021,106041.). Halloysites are 50-nm diameter biocompatible tubes with a positively charged inner lumen and a negatively charged outer layer found in nature. These features allow them to be adaptable as drug-loaded cellular membranes penetrators (Saleh et al., 2021). The Complexation of biopolymers with halloysite nanotubes (HNTs) can strongly influence their applicability as building blocks of materials (Batasheva et al., 2020). The alteration of the surfaces of Hallyosite nanotubes permits the creation of various nanoarchitectures that can enhance the thermal and mechanical efficiency of polymers and their usage for chemical controlled release (Massaro et al., 2020). The nanocomposites of halloysite and keratin protect human hair from deterioration, as evidenced by substantial cysteic acid inhibition (Cavallaro et al., 2020). Some of the most attractive nanoparticles for biomedical applications are nanoclays. According to their mineralogical composition, approximately 30 different nanoclays exist, and the more commonly used clays are bentonite, halloysite, kaolinite, laponite, and montmorillonite (Mobaraki et al., 2022).

To the authors’ knowledge, there has been little research on the impact of halloysite nanotubes on the efficacy of Asparaginase as a chemotherapeutic agent. The objective of the current study is to determine whether Halloysite nanotubes can reduce the negative impact of ASNase on ESC. In addition, their effects on histological changes and blood biochemical parameters were investigated.

## 2 Materials and Methods

### 2.1 Materials

Halloysite Nanoclay was obtained from Sigma-Aldrich Canada (685445 Aldrich), Albumin REF. K1013, Alanine aminotransferase REF. K2143, Alkaline phosphatase REF. K2115, Aspartate aminotransferase REF. K2041, Bilirubin (Total) REF. K1167 and Total protein REF. K1073 were purchased from Siemens Company, Germany.

### 2.2 Halloysite nanotubes characterization

A JEOL JEM-1230 transmission electron microscope (TEM) was used to examine the morphology of halloysite nanotubes. X-ray diffraction (XRD) patterns were performed on a Philips X-pert pro diffractometer (Ibrahim et al., 2019, Barman, 2020, Milinovic et al., 2020).

### 2.3 Experimental design

This research used 100 male adult Swiss mice weighing between 23 and 27 g, provided by the animal house at King Abdulaziz University; Jeddah, supervised by King Fahd Medical Research Center (KFMRC). The research was carried out in compliance with Canadian Ethical Standards on the Use of Trial Animals. The Rural Biomedical Ethical Committee of KAU, Jeddah, Saudi Arabia, accepted the assessment configuration. A secure lab atmosphere with a 12:12 h light: dark period, 20 °C temperature, and 65 per cent humidity. They were served a standard diet ad libitum and had free access to drinking water.

The mice were housed in plastic enclosures (25 mice per group) and held in a regulated laboratory after one week of acclimation. They were randomly allocated to four groups (25 mice each). Group 1 (control) was carried out and provided distilled water injections. ESC-bearing mice in Group 2 (ESC group) obtained an intramuscular injection (IM) of 0.15 ml Ehrlich cells (2 × 10^6^), while ESC-bearing mice in Group 3 (ESC + ASNase group) received 0.07 ml ASNase at a dosage of 6 mg/kg. Halloysite nanotubes were administered to the animals in six equivalent doses by intratumoral injection (IT) over two weeks for a total dose of 6 mg/kg. Group 4 (ESC + ASNase + HNTs) received a three-times-week intratumorally infusion of 0.07 ml ASNase (6 mg/kg) stacked on HNTs (30 mg/kg) for approximately fourteen days (Hassani et al., 2015).

### 2.4 Blood sampling

At the end of the experiment, blood was drawn from the *retro*-orbital venous plexus and placed in pure, sterile test tubes with no anticoagulants. The blood specimens were centrifuged for 10 min. At 5000 pm, the blood sera were obtained, aliquoted, and stored at − 80 °C until needed (Albooq et al., 2021).

### 2.5 Ehrlich ascites carcinoma cells

The Ehrlich ascites carcinoma cells line was kindly given at KFMRC, KAU, Jeddah, Saudi Arabia. The tumour cell line was maintained in male albino mice through serial intraperitoneal injection transplantation of Ehrlich ascites carcinoma 2.5 x10^6^ cancer cell cells/0.15 ml. Ehrlich cells were injected intramuscularly (IM) (Al-Ahmari and El-Hamidy, 2021).

### 2.6 Histopathological examination

Liver tissues were fixed in 10% formal saline for 24 h. The samples were then dehydrated using varying amounts of ethyl alcohol. Samples were cleared in xylene and embedded in paraffin for 24 h in a hot oven at 56°. Embedded in paraffin with a thickness of 5 µm were prepared for slicing using a microtome. The obtained sample parts were mounted on a microscope slide, deparaffinized, and dyed with hematoxylin and eosin stain for examination. After that, an inspection was conducted at various magnifications by a light microscope (Olympus BX 51, Olympus America, Melville, NY) and a digital camera (Salah et al., 2021).

### 2.7 Liver function parameters

Aspartate aminotransferase procedure was evaluated by a bichromatic (340–700 nm) rate method, which is an amendment of the technique established by IFCC (Wang et al., 2019). Alanine aminotransferase ALT technique used by Dimension Vista is an evolution of the IFCC suggested ALT protocol as mentioned by (Zhang et al., 2015). A bichromatic (340, 700 nm) rate method is used to measure the alteration in the absorption spectrum, which is directly related to ALT levels (Tietz et al., 2006, Shaw, 1983). Alkaline phosphatase is based on the IFCC procedure for calculating alkaline phosphatase activity at 37 °C. The system depends on utilizing dichromatic (405/700 nm) (Bowers and Mccomb, 1966, Rej, 1977). The total bilirubin technique improves the (Doumas et al., 1987) standard endpoint method. The total protein technique is an alteration of biuret reaction, which was later revised by (Kingsley, 1942). The specimen's TP level can be determined using a bichromatic spectrophotometer (540, 700 nm) endpoint technique. The albumin procedure is based on the bromocresol purple (BCP) dye-binding techniques described by (Carter, 1970, Louderback and Shanbrom, 1968).

### 2.8 Statistical analysis

The data were analyzed with IBM SPSS (IBM SPSS, IBM Corp., Armonk, NY, USA). The results were presented as mean ± standard deviation (SD). One-way analysis of variance was conducted (ANOVA) to compare different values, and the level of significance was set as P < 0.05.

## 3 Results

### 3.1 Halloysite nanoclay characterizations

**Fig. 1:**
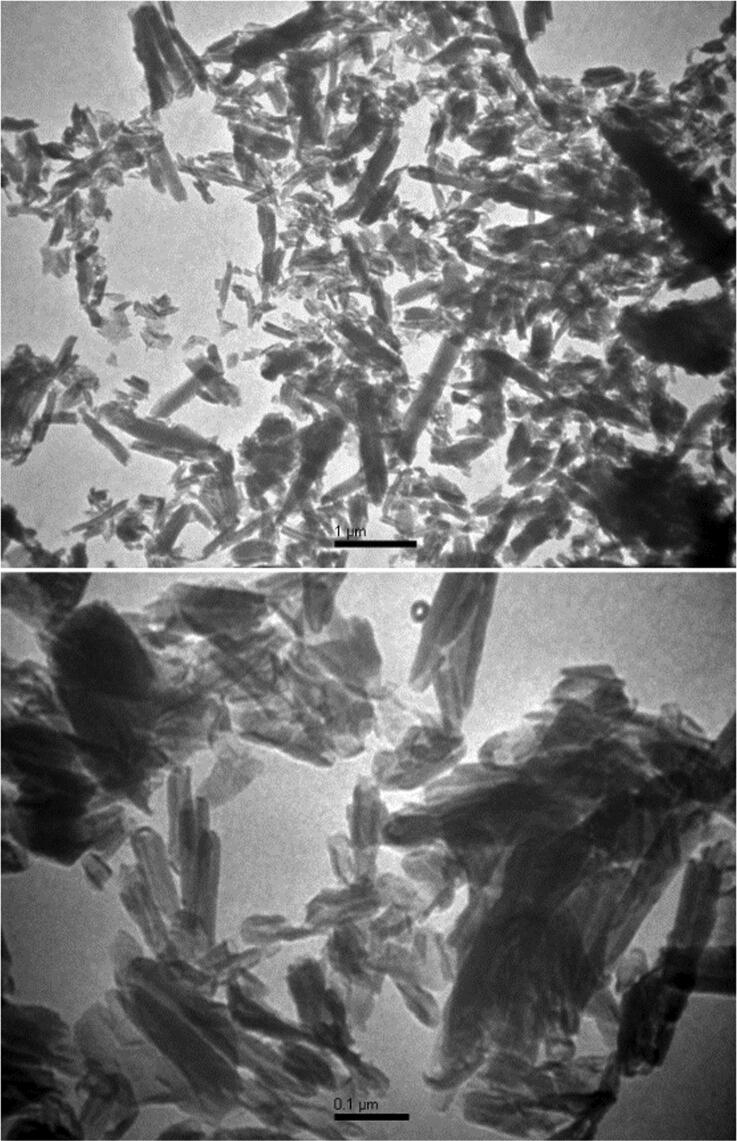
Transmission Electron Microscope micrographs of Halloysite Nanotubes at different magnification power.

**Fig. 2:**
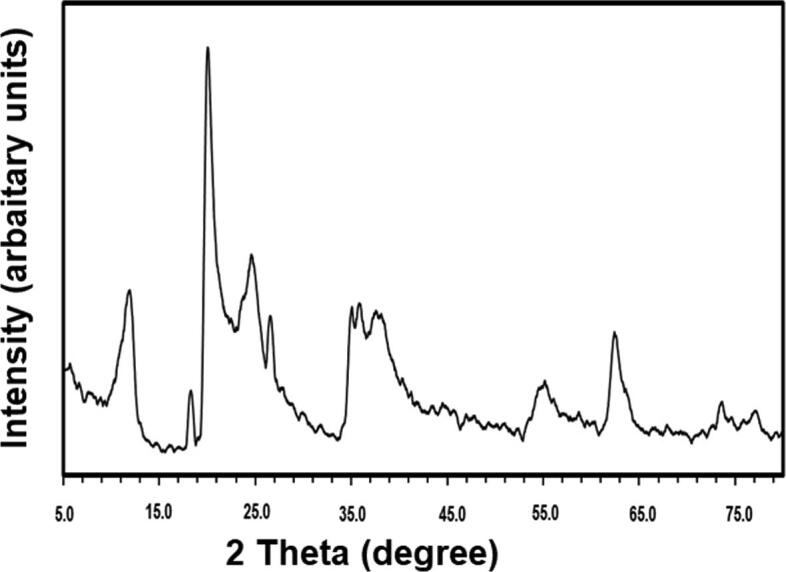
X-ray diffraction pattern of halloysite Nanoclay.

The morphology of the Halloysite nanoclay was emphasized using transmission electron microscopy. Fig. 1 shows the TEM measurements for the Halloysite nanoclay. It is noted that Halloysite nanoclay has a tubular cylindrical shape with a mean diameter of 50 nm and an average length of 1 μm. The x-ray diffraction pattern of the Halloysite nanoclay shows that the characteristic peaks were perfectly indexed to standard JCPDS file no.29–1487 (see Fig. 2).

### 3.2 Histological examination

The structure of the albino mice liver in this study is described previously.

Light micrograph of the liver of Control group mice (a) low power (X 100) to show CV; Central Vein, PV; Portal Vein, BD; Bile Duct, Hematoxylin & Eosin staining, (b) & (c) High power (400X) showing tightly packed cords of polygonal hepatocytes with rounded vesicular nuclei and acidophilic cytoplasm, bi-nucleated hepatocytes, S; The blood sinusoids, Show intact endothelial cells with flattened nuclei. Scattered irregular Kupffer cells with oval nuclei nearby hepatocytes also showed typical structure.

Light microscopic photographs of the liver of Ehrlich group mice (d) low power to show illustrating sinusoidal infiltration of carcinoma cells mixed with foci numerous polymorphs around central, portal veins and bile duct (black arrows), PV; Portal area showed large portal vein, CV; it appears in a large and irregular shape congested, dilated and surrounded by some necrotic hepatocytes (N) compared to the control group and, BD; The bile duct looked shrunk and unstable, numerous nuclei showed karyorrhexis, and there was marked nuclear pyknosis (100X), (e-f) High power to showing Inflammatory cells aggregation, diffuse necrosis of liver cells (blue arrows), vascular endothelial cell rupture (X400).

Light microscopic photographs of paraffin sections of the liver of Ehrlich + Asparaginase group mice (g) low magnification notice an improvement in shape and size of the central vein. There were some blood cells in the portal veins, mild inflammatory cells (white arrow), and bile ducts are become regular in shape (100X), (h-i) High magnification to show moderate cell necrosis and tumour cell than the Ehrlich group, bi-nucleated hepatocytes (Red arrow) and Portal vein filled with blood cells.

**Fig. 3:**
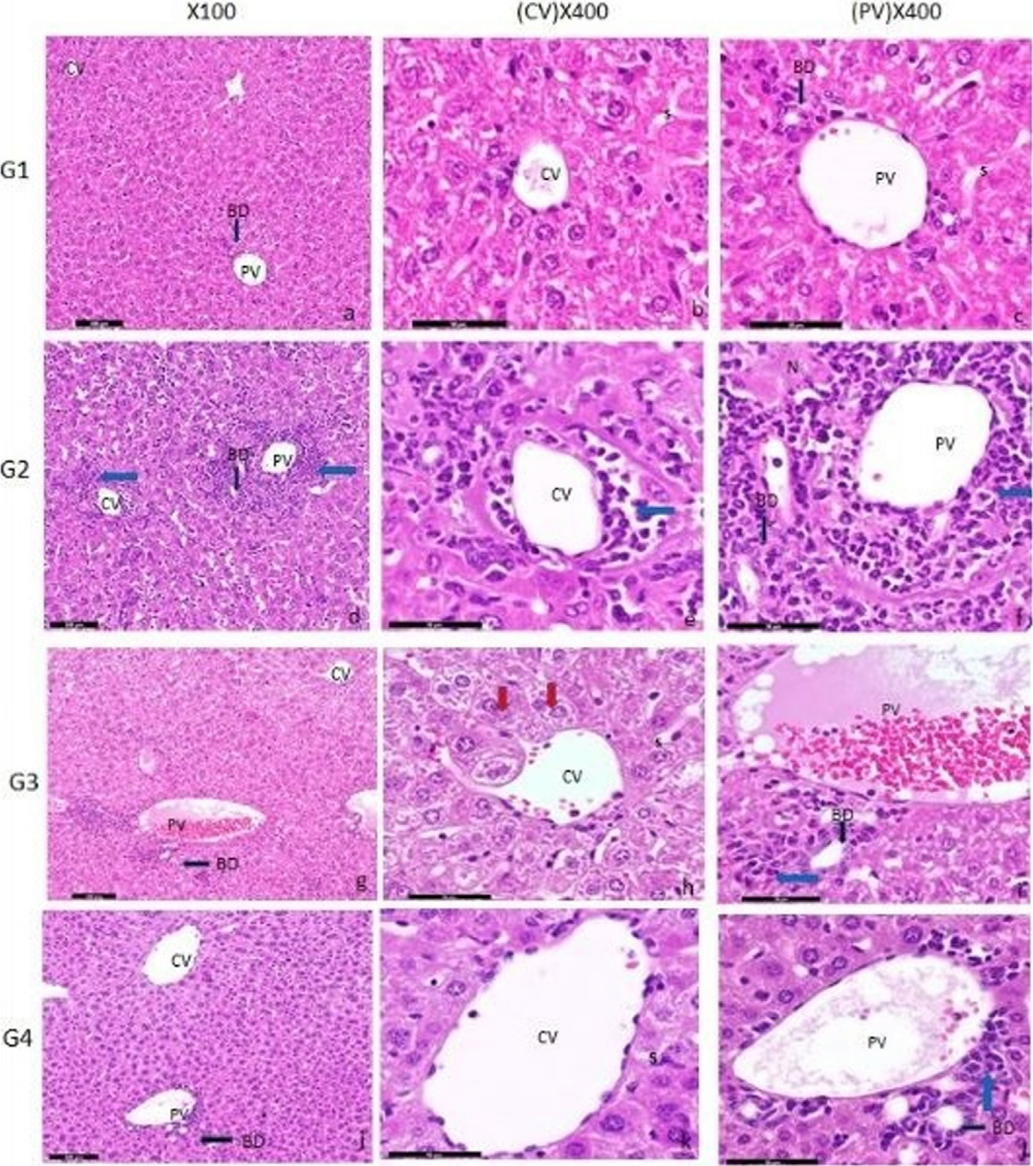
Mice liver stained sections by Hematoxylin and eosin were photographed at different magnification powers (X100 & X400) for G1, G2, G3, and G4.

A photomicrograph of a section in the liver of adult albino mice of Ehrilch + Asparginase + Halloysite nanotubes group (j) low power (100X) showing absence of tumour cells around the central and portal veins or inside the cells, central and portal veins become regular in shape and size. There is an improvement in the form of the hepatocytes with an expansion in size steady in the shape of the bile duct mild inflammatory cells (blue arrow). (k-l) High power (X400) to show less cell necrosis, Hepatic cells with colloidal cytoplasm contain inclusions (Fig. 3).

### 3.3 Liver functions

Liver functions were significantly increased in ESC and ESC + ASNase groups versus the control group (*P* < 0.0001 for both). ALT concentrations were significantly decreased in ESC + ASNase, ESC + ASNase + HNTs groups versus ESC group (*P* < 0.0001 for all); in ESC + ASNase + HNTs group versus ESC + ASNase group (*P* < 0.0001). Serum levels of AST were significantly increased in ESC, ESC + ASNase, and ESC + ASNase + HNTs groups versus the control group (*P* < 0.0001). AST levels were significantly decreased in ESC + ASNase, ESC + ASNase + HNTs group versus ESC group (*P* < 0.0001 for all); in ESC + ASNase + HNTs.

**Fig. 4:**
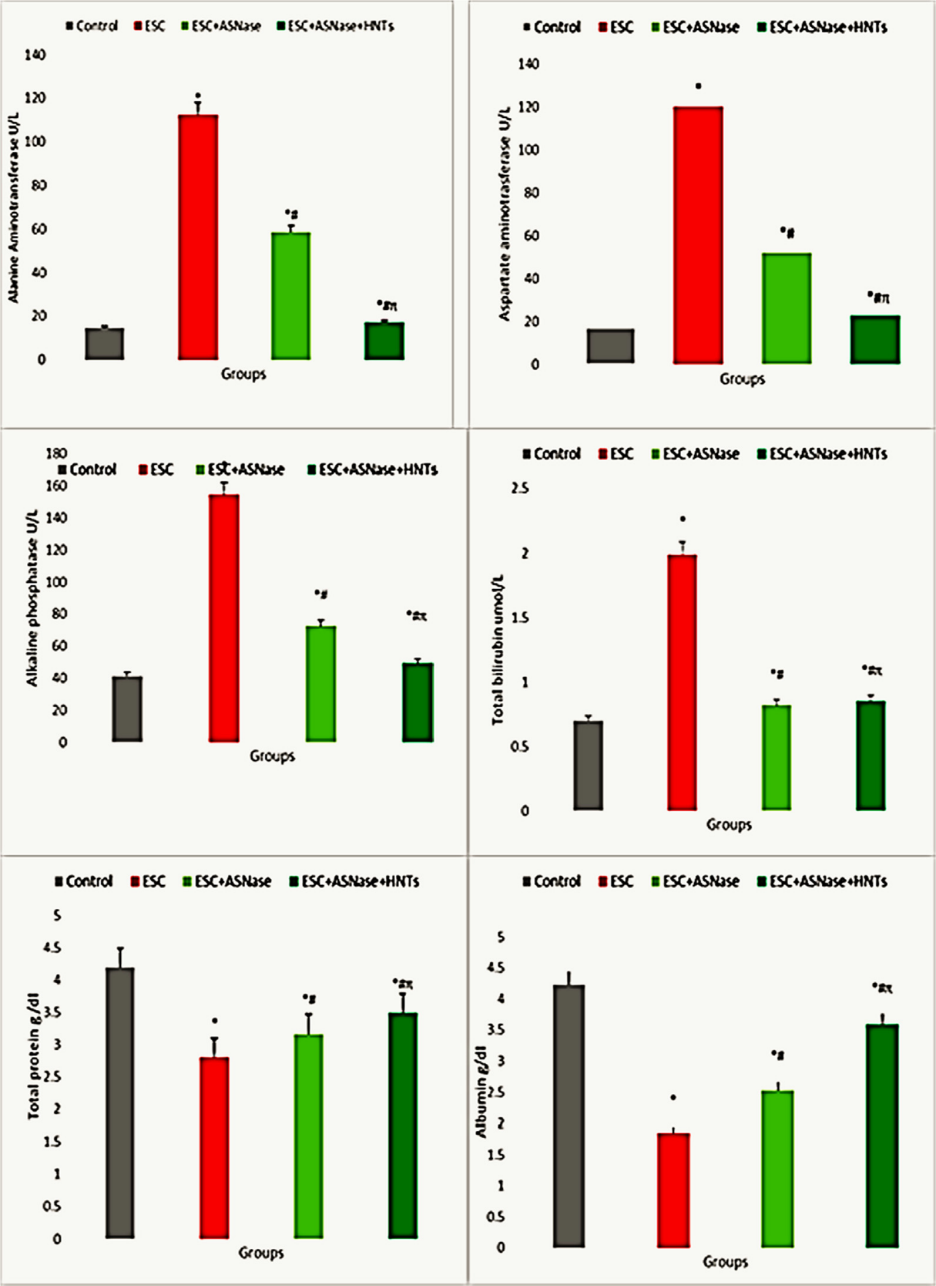
Difference between all studied groups among liver functions in adult male albino mice.

Serum levels of ALP were significantly increased in ESC, ESC + ASNase and ESC + ASNase + HNTs groups versus the control group (*P* < 0.0001). ALP levels were significantly decreased in ESC + ASNase, ESC + ASNase + HNTs and ESC + ASNase + HNTs-CS groups versus ESC group (*P* < 0.0001 for all); in ESC + ASNase + HNTs and ESC + ASNase + HNTs-CS groups versus ESC + ASNase group (*P* < 0.0001) and in ESC + ASNase + HNTs-CS group versus ESC + ASNase + HNTs group (*P* < 0.0001). Serum levels of total bilirubin were significantly increased in ESC, ESC + ASNase and ESC + ASNase + HNTs groups versus the control group (*P* < 0.0001, *P* = 0.008 and *P* = 0.002). Total bilirubin levels were significantly decreased in ESC + ASNase and ESC + ASNase + HNTs groups versus ESC group (*P* < 0.0001 for both). Total protein and albumin levels were significantly decreased in ESC and ESC + ASNase, ESC + ASNase + HNTs groups versus the control group (*P* < 0.0001 for all). Total protein and albumin levels were significantly increased in ESC + ASNase and ESC + ASNase + HNTs groups versus ESC group (*P* < 0.0001 for all); in ESC + ASNase + HNTs group versus ESC + ASNase group (*P* < 0.0001) (Table 1 and Fig. 4).

**Table 1:** Difference between all studied groups among liver functions in adult male albino mice.

Groups	AST U/L	ALT U/L	ALP U/L	TBIL umol/L	TP g/dl	ALB g/dl
Control	17.25 ± 0.25	14.75 ± 0.25	41.25 ± 0.25	0.70 ± 0.09	4.20 ± 0.05	4.23 ± 0.25
ESC	120.58 ± 0.63*	157.25 ± 0.25*	154.50 ± 0.43*	1.99 ± 0.01 *	2.80 ± 0.05*	1.85 ± 0.10*
ESC + ASNase	52.20 ± 1.87^*#^	58.70 ± 0.20^*#^	72.50 ± 0.25 ^*#^	0.82 ± 0.01^*#^	3.17 ± 0.14^*#^	2.53 ± 0.20^*#^
ESC + ASNase + HNTs	23.75 ± 0.75^#π^	14.25 ± 0.25^*# π^	49.33 ± 0.38^*# π^	0.85 ± 0.01^*#^	3.50 ± 0.25^*# π^	3.59 ± 0.04^*# π^

Values are expressed as mean ± standard deviation. *: Significant change compared to the control group using ANOVA-one way (LSD) at P ≤ 0.05. #: Significant change compared to the Ehrlich Solid Carcinoma group using ANOVA-one-way (LSD) P ≤ 0.05. π: Significant change compared to the Ehrlich Solid Carcinoma and Asparaginase group by using ANOVA-one way (LSD) at P ≤ 0.05.

ESC: Ehrlich Solid Carcinoma, ESC + ASNase: Ehrlich Solid Carcinoma + Asparaginase and ESC + ASNase + HNTs: Ehrlich Solid Carcinoma + Asparaginase + Halloysite nanotube. AST: Aspartate Aminotransferase. ALT: Alanine Aminotransferase. ALP: Alkaline Phosphatase. TBIL: Total bilirubin. TP: Total protein. ALB: Albumin.

## 4 Discussion

The structure and morphological shape of the halloysite nanotubes have been emphasized using the TEM and XRD measurements. The TEM images revealed that the halloysite nanoclay has a tubular cylindrical shape with a mean diameter of 50 nm and an average length of 1 µm.

The XRD measurements of the Halloysite nanoclay showed diffraction patterns at 12.17°, 20.11°, 24.69°, 35.10°, 38.46°, 54.69°, 62.45°, 73.75°, and 77.09°, which matched well the diffraction from the halloysite powder (JCPDS file no. 29–1487) (Gaaz et al., 2017, Bianchi-Frias et al., 2019).

Ehrlich's melanoma has powerful inflammatory occurrences, such as ischemia and inflammatory infects, which are assumed to significantly affect cancer development in several kinds of malignancy (Unsoy et al., 2014). Nanotechnology has been used to enhance therapy for cancer by increasing the bioavailability and medicinal uses of anticancer drugs. Nanotechnology's application in cancer treatment has an important implication, such as the potential to remove cancers with limited losses to normal tissue using novel targeted therapeutics (Bertolino et al., 2016).

The potential of halloysite nanotubes to converse with medications and molecules for targeted release, and their aptitude to connect with polymers like chitosan for excellent mechanical, has sparked a surge in benefit in clays' use in biomaterial architecture. As a result, an increasing study has found clay's bioactivity and directly interact with cells and tissues.

Because of one's low price, high accessibility, high exchangeable cations, high absorption ability, and large surface area, nanoclays have been the most used synthetic elements in preparing nanocomposites (Depan et al., 2009). Although there is no literature regarding eliminating halloysite nanotubes from the body, other studies may suggest different pathways for removing nanoparticles from the body, hepatobiliary elimination (Hu et al., 2017), and renal elimination (Poon et al., 2019) because the nephron is helpful for the quick elimination of molecules from the intravascular space, mainly in their administered forms. Kidney clearance is the desired mechanism for nanoparticles or nanotubes elimination from the body with minimum catabolism to prevent adverse reactions (Keliher et al., 2017).

The severe symptoms of solid tumours, which include injury tissue such as the heart, liver, and spleen, are significant issues. Chemotherapeutic drug hepatotoxic impacts can consist of fibrosis, steatosis, necrosis, hypoglycemia, and vascular injury, in addition to liver damage (Michelle et al., 2008).

Liver illnesses have become the primary cause of death. Chemical-induced tissue damage is among the most frequent types, and it poses severe therapeutic and regulatory challenges. Due to the use of numerous drugs and being affected by multiple toxic substances, hepatic harm has been a significant health issue (Zargar et al., 2017).

The findings showed elevated serum levels of AST, ALT, ALP, and TBIL in the ESC treated patients compared to controls, reducing total protein and albumin. Additionally, after ASP therapy, ESC + ASP + HNTs group, the concentrations of these variables decreased. The AST enzyme is used to diagnose hepatic disabilities, and a rise in its activity indicates severe liver failure and hepatic illnesses. ALT is a critical and valuable diagnostic tool for liver damage. ALT is more precise for liver disease than AST in symptoms of liver injury. Since these enzymes are cytoplasmic, when the liver is injured, the absorption of the hepatic plasma membrane is changed, allowed to enter the vascular system (Kober et al., 2016).

EAC inoculation significantly increased serum levels of AST, ALT, and total bilirubin in mice. Still, serum total protein and albumin levels significantly decreased compared to control, implying an impaired liver function. (Kapoor et al., 2014). Total protein changes caused in cirrhotic livers interruption in carbohydrate, protein, and lipid metabolic rates, as well as the incensed protein biosynthesis (Amin et al., 2012). Obstruction of bile ducts caused by stress and thrombosis in portal triads and repetition of diverticular disease from fibrotic liver cells to ventricles may cause increased bilirubin levels (Algandaby, 2018). Enhanced synthesis in rising biliary pressure causes an increase in serum ALP (Poupon, 2015).

According to a wealth of data, the reduced total protein content is attributed to the role in the progression of liver injury abnormalities (Esmaili et al., 2014, Junior et al., 2016) exposed that albumin levels in the EAC-bearing mice were significantly reduced (Mesalam, 2013). The control group had a typical liver structure with no histopathological lesions. Liver sections from mice with Ehrlich ascites carcinoma showed vacuolization of hepatic cytoplasm, irregular cell necrosis of individual liver cells with highly pyknotic nuclei, congestion of the central vein connected with infiltrated cells, and an area of hemorrhage, among other histopathological alterations.

Hepatocytes in treated mice with ANase degenerated, with karyolysis and pyknosis of the nuclei. Also seen were a swollen central vein with hemorrhage, constipated blood sinusoids, and monocyte invasion (Alshaymaa et al., 2012).

Typically, liver failure caused by tumour cells signifies an interruption of the metabolic processes of liver cells due to alterations in serum enzymatic activity. A significant rise in serum levels of ALT, AST and ALP were observed in the present study, together with a consequent decrease in serum albumin and total protein levels in ESC-bearing mice, indicating liver damage. The rise in liver enzymes may result from the generalized damage of hepatocytes and the leak into the plasma of AST following tumour activation. Albumin, the most abundant plasma protein, and other plasma proteins are produced in liver hepatocytes and are essential biomarkers of liver function that can be considered reliable (Saravanan et al., 2006). The significant decrease in serum albumin and total protein levels monitored in mice with Ehrlich solid tumours is equivalent to liver failure, manifested as decreased biosynthetic capacity.

EAC caused ALT, AST, ALP elevations, albumin, and total protein reduction. Our results showed that liver enzyme elevation indicates hepatic depletion associated with cancer prevalence (El-Atrsh et al., 2020). According to the literary investigation, the growth of Ehrlich's cancer in mice was caused by changes in the liver (El-Masry et al., 2019, Donia et al., 2018, Ahmed et al., 2019).

In the present work, there was an important elevate in serum rates of ALP, ALT, and AST, along with a subsequent decrease in albumin and total protein levels in ESC-bearing mice, demonstrating liver failure. The elevation in liver enzymes may result from generalized hepatocyte injury and leakage of AST into the plasma after tumour modulation. In liver hepatocytes, albumin, the most incredible abundant plasma protein, and other plasma proteins are produced and are significant liver function biomarkers that can be considered reliable. Therefore, the substantial reduction in serum albumin and total protein levels observed in Ehrlich solid tumour mice is equal to liver cancer, which reduces biosynthetic efficiency. Asparaginase has been commonly used as an anticancer drug, and the used halloysite nanotubes as drug delivery system enhancement in the efficiency of Asparaginase showed the anticancer potential of the drug.

Author Contributions.

B.M.M.B is a student of the Master in Biological Sciences Dept., Faculty of Sciences, King Abdulaziz University, Jeddah, Saudi Arabia and has contributed equally in collecting and cross-checking the data; S.A.M., M.A., and S.M.E. were involved in the literature search and writing of the original draft of the introduction; H.A., N.A.A., and A.H.B. have conceptualized, analyzed the data, written, and made the scientific review; M.A. and S.M.E. editing of the final version of the paper. All authors have read and agreed to the published version of the manuscript.

## Funding

This research received no funding.

## Declaration of Competing Interest

The authors declare that they have no known competing financial interests or personal relationships that could have appeared to influence the work reported in this paper.
